# A retrospective observational study analyzing work and study motivation based on the work environment of 15,677 Japanese clinicians in 2016

**DOI:** 10.1038/s41598-022-19007-9

**Published:** 2022-08-31

**Authors:** Yuzo Shimazu, Yurie Kobashi, Seiya Imoto, Masaharu Tsubokura

**Affiliations:** 1 Department of Radiation Health Management, Fukushima Medical University School of Medicine, Fukushima, Fukushima 960-1295 Japan; 2 Division of Health Medical Intelligence, Human Genome Center, The Institute of Medical Science, The University of Tokyo, Tokyo, Japan

**Keywords:** Health care, Health occupations

## Abstract

Physicians play an active role in public health. However, there is a limit to the knowledge and experience that can be gained through hospital work alone. This was a secondary data analysis from 100,000 doctors in Japan (15,677 respondents). The results of the analysis showed that 898 (8.4%) male and 190 (6.0%) female doctors worked 60 h or more in a week. The percentage of physicians whose spouse was a physician was found to be 31.4% (male) and 61.7% (female) (p < 0.001), and the rate of full-time working clinicians was 85.7% (male) and 30.0% (female) (p < 0.001). In the univariate analysis, female’s working hours were affected by childbirth and childcare experience (p < 0.001, 95% CI − 10.3 to − 8.4, with “none” as reference) and specialty certification (p < 0.001, 95% CI − 3.5 to − 1.4, with “none” as reference). In the multivariate analysis, physician’s working hours were associated with sex (coefficient, − 7.4; 95% CI − 8.3 to − 6.5, with “male as reference), childbirth/childcare (coefficient, − 2.2; 95% CI − 2.9 to − 1.4, with “possession” as reference), and specialty qualification (coefficient − 4.0, 95% CI − 5.0 to − 3.0, with “possession” as reference). To summarize the results of the analysis, work/study motivation of physicians will be facilitated by ensuring adequate learning opportunities and by developing support systems and environments.

## Introduction

Health care professionals must acquire not only knowledge and skills, but also the ability to collaborate with other professionals^1,2^. For them to acquire these abilities, it is important that they continue to learn throughout their lives and develop a broad career. In recent years, as social conditions and values have become more diverse, physicians are expected to play a wide range of roles not only within the hospital but also in society outside the hospital. For example, during a global pandemic^3,4^, it is important for medical personnel to take a comprehensive view of society not only from a medical perspective, and to take a leadership role in solving public health problems^5^. To acquire such abilities, young physicians need to receive a well-rounded post-graduate education and learn to build a foundation for a lifelong career^6,7^. Professionals need to ensure adequate learning based on work hours (clinical office hours, non-clinical office hours, on-call hours, etc.) and off-job hours^1,8^.

However, there is no clear global consensus on the amount of time required for residents to acquire skills^9^. This is because there are differences in the maximum weekly working hours for residents in European and North American countries. The difference ranges between 48 and 80 h. Residency programs in European countries show good compliance with this standard. However, less than 25% of European Union member states successfully apply this standard. This result suggests that many issues remain to be resolved, such as the reorganization of health services and labor practices, if countries are to meet the appropriate standard for working hours^10,11^. Particularly, there is a global shortage of doctors^12^. The working hours of doctors include long hours due to the addition of on-call hours^13,14^. Physicians are expected to have medical knowledge and skills, communication skills, and the ability to look at society, regardless of on-job training or off-job training^15^. Currently, a study of the relationship between physicians' working hours and study time has not revealed any factor that leads to career advancement.

To gain an accurate understanding of the current working patterns of physicians, their work style intentions, and career awareness, a survey of the working conditions and work style intentions of 100,000 physicians was conducted in December 2016 in Japan. While there is little information on the working intention of physicians globally, the strength of this survey is the large number of responses received and the included questionnaire on their living and working conditions, as well as their future intentions. The analysis of such data is very useful in discussing the employment status and work/study motivation of doctors.

Therefore, we conducted an observational study of physicians' working hours based on secondary data obtained from the survey to elucidate the factors necessary for employment status and work/study motivation.

## Methods

This study is a secondary data analysis of the physician work style survey conducted in 2016.

### The physicians’ work style survey conducted in 2016

In the survey, firstly, the medical facilities in Japan were stratified into four groups by the number of the hospital beds (199 beds or less, 200–399 beds, 400–799 beds, and 800 beds or more or special function hospitals). Accordingly, there were a total of 12,035 medical facilities (11,319, 499, 195, and 22 facilities, respectively) that were randomly selected from each of the four groups for this survey. All physicians (full-time and part-time) working at the selected facilities were included in the survey. Those who received more than one questionnaire were asked to respond at the facility where they worked primarily. Therefore, it was hard to count the actual number of questionnaires distributed; however, approximately 100,000 questionnaires were brought to the facilities. It was predicted that the response rate would be approximately 10–20%.

A self-administered survey questionnaire method was used for the physicians. To protect the privacy of the physicians who responded, each physician sent his or her answers directly to the researcher in a collection envelope. The medical institution form was filled out anonymously by the person in charge of each facility.

Overall, 15,677 physicians from 3126 facilities responded to the survey; the response rates were 15.7% (if the total number of distributed questionnaires for physicians is 100 thousand) and 26.0%, respectively.

### Survey details of the physicians’ work style survey in 2016

First, the physician survey questionnaire covered the following four parts. The first part was composed of the physician’s attributes include the attributes asked for included: age; sex; university of origin; year of graduation; place of residence; place of birth; type of work; income; family members living together; department; specialist qualification; childbirth, childcare experience, and leave of absence due to that; and affiliation with university medical office. This item was used to extract detailed attributes to obtain an accurate picture of the current status of physicians' work styles and working conditions. The second part contained working conditions in a time schedule-study mean. To investigate the working conditions of doctors, we asked them to describe their working conditions in a timetable. The subjects described their actual work, especially during the week from Thursday, December 8 to Wednesday, December 14. Respondents had the option of choosing either “workday” or “holiday”. If they chose “holiday”, there were no responses in the table for that day. Working hours were classified into four categories: “clinical office hours”, “non-clinical office hours”, “on-call hours”, and “break time”. For the definition of terms, clinical office hours were defined as the time spent in outpatient, inpatient, and home nursing care. Non-clinical office hours were defined as time dedicated to teaching, research, self-training, meetings, and administrative duties. On-call hours were defined as the time when the physician maintains a cell phone for contact from nighttime to the next morning and treats patients when the need for medical care arises. Finally, break time was defined as time allocated for meals and resting. The third part was composed of work shared with other professions or physicians, which included work that other professionals could perform on their behalf as follows: (1) explaining procedures to patients and obtaining consent, (2) taking vital parameters and obtaining data, (3) preparing medical records, (4) medical administration, and (5) moving and transporting patients. In reality, physicians may spend a lot of time performing these tasks on their own. Therefore, to enable work sharing with other professionals, physicians were asked to indicate the actual time spent on these tasks and the percentage of time that could be shared with other professionals. These included career awareness and future work style (clinician, general practitioner, nursing care and welfare field, occupational health, administrative position, private company, research, international health, etc.). The intent of these questions was to investigate physicians' intentions regarding career development and job diversity. The fourth part included intentions and years of working in the local area. We defined urban and metropolitan areas as cities like Tokyo, government-designated cities, and places where prefectural governments are located. Other cities and towns were classified as rural areas. The medical facility questionnaire survey covered the following four parts. The first part contained attributes of the medical facility: type of establishment, type of facility, type of hospital, number of inpatients, and number of outpatients. The second part included the number of staff members working at the facility. The third part contained questions in balancing work and family, measures instated by the institution to ensure that support, and availability of support for doctors to balance work and family life at each facility. The number of doctors that took childcare leave was assessed. Questions on the availability of day-care centers were also asked, their opening hours, and their working days.

### Analysis

**Figure 1:**
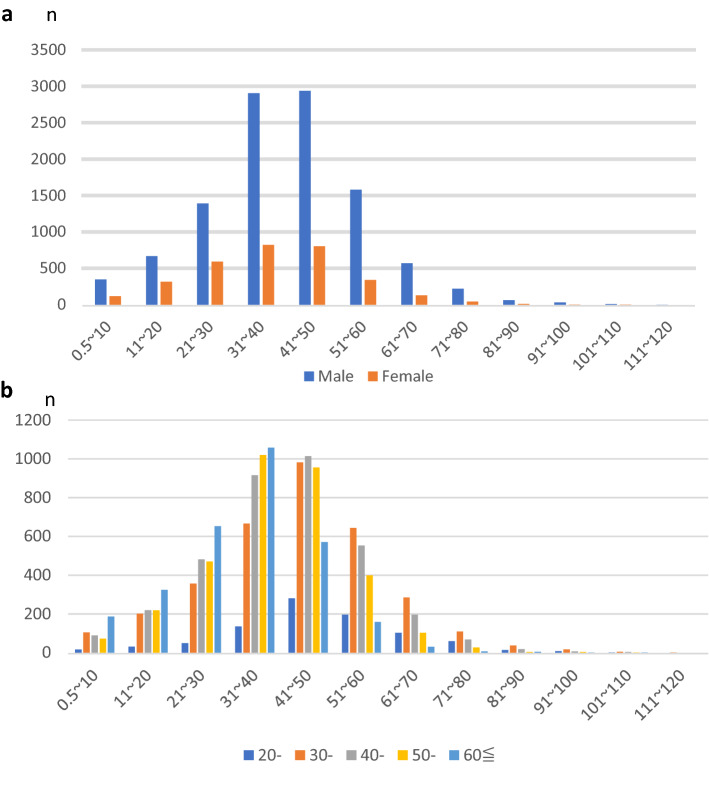
(**a**) The number of participants each weekly working hour by sex (n = 13,909). (**b**) The number of participants each weekly working hour by age (n = 14,142).

To create a database for the analysis, we matched the questionnaires for physicians and facilities using the zip code. First, we extracted all generations of doctors whose zip codes matched those of the questionnaires for doctors and medical facilities. While the basic attributes of young doctors were the crux of the discussion on lifelong career development, this study aimed to analyze the factors necessary for career development from a survey on the working styles of young doctors (men and women under 40 years of age). The chi-square test was used to analyze the differences of attributes between male and female. Secondly, to grasp the relationship between the work attributes of young doctors and the working hours of the elderly (greater than 40), the distribution of working hours by sex and age is shown as a bar graph (Fig. 1). We compiled a table of median (range) and mean values for clinical office hours, non-clinical office hours, on-call hours, and career aspirations by sex and age (Table 2). To find factors affecting physicians' working hours, we performed a univariate analysis. First, the univariate analysis showed that age, sex, department, childbirth/childcare experience, and specialty status were significantly associated with working hours.

Secondly, we attempted to construct a multivariate model. However, spousal work status and number of hospital beds could not be entered as explanatory variables due to many missing values. Therefore, we conducted a multivariate analysis using age, sex, medical department, childbirth/childcare, and whether the patient obtained a medical specialty, which were significant in the univariate analysis. The results of the multivariate regression for each type of working hours (i.e., clinical office hours, non-clinical office hours, and on-call hours) are shown in Table 3. As an appendix analysis, we analyzed efforts to continue working without leaving the job during childbirth or childcare, depending on whether the respondent has experienced childbirth or childcare (the results by sex are attached as a supplemental file). All analyses were performed using Stata/IC version 15.0 (Stata Corp, College Station, TX, USA). p-values < 0.05 were considered statistically significant.

### Ethics

This study was approved by the Ethics Committee of the Institute of Medical Science, University of Tokyo (approval number: 2020-33) and by the Ethics Committee of Fukushima Medical University (approval number: General 2020-166).

Informed consent was obtained from all the participants in the study in the form of the opt-out methods.

## Results

The overall response rate generally reflects the age distribution of the physicians.

These data are the accurate reflection of the overall employment situation of physicians in Japan. Therefore, from the attributes of physicians of all ages, we can extract the working styles and environment surrounding their employment. Consequently, we were able to clearly describe the differences in career development by sex.

We cross-referenced the physician questionnaire with the medical facility questionnaire based on their zip codes and selected 12,293 physicians. We sampled physicians under the age of 40 to describe the work styles of younger physicians and the environment surrounding their employment. The results showed that a total of 5094 (41.4%) were under 40 years (3295 males, 64.7%; 1799 females, 35.3%).

Table 1 shows the basic demographics of the younger generation of physicians aged < 40 years. The number of valid responses varied by item. A total of 695 respondents provided their spouses’ occupations. A total of 282 respondents had partners who were doctors (153 males, 31.4%; and 129 females, 61.7%; respectively p < 0.001), with a statistically significant difference. Additionally, 318 respondents (144 males, 30.0%; and 174 females, 85.7%) had spouses who worked full time, and the difference was statistically significant (p < 0.001).

**Table 1:** Characteristics of physicians under 40 years by sex (n = 5094).

	Male		Female		Total	p value*
	n	%	n	%		
**Age category, years**	3295		1799		5094	p < 0.001**
26–30	807	24.4	524	29.1	1331	
31–40	2488	75.5	1275	70.9	3763	
Hospital type	2259		1232		3491	p = 0.22
**Training core**	11	0.5	9	0.7	20	
Cooperation	79	3.5	39	3.2	118	
Specialized training	119	5.3	57	4.6	176	
Emergency designation	39	1.7	24	1.9	63	
Secondary emergency	494	21.9	231	18.8	725	
Emergency and critical care center	119	5.3	80	6.5	199	
Maternal and perinatal center	1195	52.9	665	54.0	1860	
Support at home	203	9.0	127	10.3	330	
**Work style**	2230		1226		3456	p < 0.05***
Full time	1771	79.4	907	74.0	2657	
Part time	459	20.6	319	26.0	778	
**System of attending physician**	2227		1218		3445	p < 0.001**
Single	1016	45.6	546	44.8	1562	
Multiple	939	42.2	450	36.9	1389	
Others	272	12.2	222	18.2	494	
**On-call at night**	2139		1103		3242	p < 0.001**
On call	968	45.3	423	38.3	1391	
Shift	1171	54.7	680	61.7	1851	
**Workplace**	1111		637		1748	p = 0.96
Urban and metropolitan	835	75.2	478	75.0	1313	
Rural	276	24.8	159	25.0	435	
**Department**	813		458		1271	p < 0.001**
Internal medicine	335	41.2	181	39.5	516	
Surgeon	152	18.7	25	5.5	177	
Obstetrics and gynecology	23	2.8	46	10.0	69	
Pediatrics	66	8.1	59	12.9	125	
Emergency	22	2.7	7	1.5	29	
Anesthesiology	35	4.3	51	11.1	86	
Psychiatry	73	9.0	30	6.6	103	
Radiology	35	4.3	18	0.2	53	
Resident	72	8.9	41	9.0	113	
**Beds**	779		437		1216	p = 0.1
0–19	1	0.1	1	0.2	2	
20–49	3	0.3	3	0.7	6	
50–100	17	2.2	9	2.1	26	
101–200	65	8.3	39	8.9	104	
201–300	60	7.7	34	7.8	94	
301–400	58	7.4	33	7.6	91	
401–500	51	6.5	52	11.9	103	
501–	524	67.2	266	60.9	790	
**Income**	779		431		1210	p < 0.001**
≤ 500	80	10.3	87	20.2	167	
501–1000	293	37.6	221	51.3	514	
1001–1500	287	36.8	104	24.1	391	
1501–2000	101	13.0	15	3.5	116	
≥ 2001	18	2.3	4	0.9%	22	
**Specialist qualification**	730		408		1138	p = 0.10
Possession	423	57.9	216	52.9	639	
None	307	42.1	192	47.1	499	
**Annual vacation (days)**	697		389		1086	p = 0.19
0	215	30.8	110	28.3	325	
1–5	327	46.9	181	46.5	508	
6–10	135	19.4	77	19.8	212	
11–	20	2.9	21	5.4%	41	
**University medical department**	683		383		1066	p = 0.19
With affiliation	550	80.5	291	76.0	841	
Planned affiliation	54	7.9	34	8.9	88	
No affiliation planned	79	11.6	58	15.1	137	
**Partner job**	486		209		695	p < 0.001**
Physician	153	31.4	129	61.7	282	
Medical stuff	170	35.0	13	6.2	183	
Non-medical	163	33.5	67	32.1	230	
**Partner's work style**	480		203		683	p < 0.001**
Full-time employment	144	30.0	174	85.7	318	
Part-time employment	113	23.5	19	9.4	132	
Self-employed	3	0.60	7	3.4	10	
Unemployed	220	45.8	3	1.5	223	
**Children**	480		203		683	p < 0.001**
None	117	24.4	82	40.4	199	
Possession	363	75.6	121	59.6	484	

*p-values for the chi-square test for comparisons between male physicians and female physicians.

**p < 0.001, ***p < 0.05.

Given the differences in the attributes of young doctors, we summarized the distribution of working hours for all generations in the figures. Figure 1a shows the distribution of weekly working hours by sex (n = 13,909). The most frequent response was “41–50 h” (n = 3743 [26.9%]), with a difference between males and females of 2939 [27.4%] and 823 [25.9%], respectively (p < 0.05). A total of 898 (8.4%) males and 190 (6.0%) females were overworked for more than 60 h, while 108 males (1.0%) and 18 females (0.5%) (n = 135, 10.0%) worked more than 81 h per week. Figure 1b showed the distribution of age and working hours (n = 14,142). The highest frequency was 31–40 working hours (n = 3803); Regarding overworking, 1116 people (29.3%) worked ≥ 60 h and 128 people (3.4%) worked ≥ 81 h (2.3%).

Table 2 summarized the details of clinical office hours and on-call hours (n = 15,337; 11,762 males, 76.7%; 3575 females, 23.3%). The overall median clinical office hours was 40 (range, 0.5–119) hours, 41 (range, 0.5–119) for males and 37.5 (range, 2–107) for females. There was no clear difference in the time spent on miscellaneous duties between age and sex. As for future career preferences, there was a shift from clinical to private practice. There was no significant difference in the desire to increase or decrease workload between the age or sex groups.

**Table 2:** Work hours for December 8–14, 2016, work shared with other professions, and future intentions (n = 15,379).

	Male			Female			Overall		
	Min	Max	Median	Min	Max	Median	Min	Max	Median
**Clinical office hours (h)**									
All ages, years	0.5	119.0	41.0	2.0	107	37.5	0.5	119.0	40.0
20–	3.0	104.5	49.5	2.0	90	47.5	2.0	104.5	48.5
30–	1.0	119.0	46.5	3.0	101	39.5	1.0	119.0	44.0
40–	1.0	109.5	43.5	2.0	100	34.0	1.0	109.5	41.0
50–	0.5	102.5	40.0	2.0	88	35.8	0.5	102.5	39.5
≥ 60	0.5	96.0	34.0	4.5	107	32.5	0.5	107.0	34.0
**On-call hours (h)**									
All ages, years	0.5	119.0	41.0	0	126.5	0	0	127.0	0
20–	3.0	104.5	49.5	0	93.5	9.0	0	114.0	12.0
30–	1.0	119.0	46.5	0	126.5	0	0	126.5	8.5
40–	1.0	109.5	43.5	0	123	0	0	127.0	0
50–	0.5	102.5	40.0	0	103	0	0	126.0	0
≥ 60	0.5	96.0	34.0	0	93	0	0	122.0	0

Number and percentage of employees on call									
	Male			Female			Overall		
	n	%	Total	n	%	Total	n	%	Total
All ages, years	1342	38.6	3471	313	27.9	1122	1679	36.0	4668
20–	128	57.9	221	61	45.2	135	190	52.9	359
30–	442	58.7	753	143	36.1	396	592	50.9	1164
40–	348	48.6	716	69	23.2	298	421	40.8	1032
50–	250	32.1	777	30	17.2	174	289	29.8	971
≥ 60	174	17.3	1004	10	8.4	119	187	25.3	1142

Time actually spent on business on 12/14									
	Male			Female			Overall		
**Patient explanation/consent acquisition (min)**									
All ages, years	1	960	60	1	660	85	1	960	60
20–	5	770	60	8	480	60	5	770	60
30–	5	840	60	5	600	60	5	840	60
40–	1	960	60	1	660	90	5	960	60
50–	1	660	90	5	570	100	1	660	80
≥ 60	3	750	100	10	600	120	3	750	90
**Data management (min)**									
All ages, years	1	960	30	1	720	30	1	960	30
20–	1	660	30	1	360	30	1	660	30
30–	1	960	30	1	720	30	1	960	30
40–	4	600	30	1	600	30	1	600	30
50–	1	630	40	5	540	40	1	630	40
60–	2	750	50	5	420	40	2	750	50
**Medical records (min)**									
All ages, years	3	720	90	2	720	90	2	720	90
20–	10	480	90	10	630	100	15	630	100
30–	3	720	90	3	720	80	5	720	60
40–	5	660	90	2	660	90	2	630	80
50–	10	500	90	3	600	90	3	600	90
60–	10	480	110	2	600	80	2	600	75
**Office work (min)**									
All ages, years	2	480	30	1	600	30	1	600	30
20–	5	300	40	5	360	50	5	360	50
30–	3	480	30	3	480	30	3	420	30
40–	5	420	30	1	600	30	1	600	30
50–	2	390	30	2	420	30	2	420	30
≥ 60	10	410	30	5	480	30	5	480	30
**Patient transfer and goods movement (min)**									
All ages, years	2	420	20	1	700	30	1	700	30
20–	5	120	30	1	400	30	1	400	30
30–	2	180	20	1	300	30	1	300	30
40–	5	420	17.5	2	700	20	2	700	25
50–	2	90	15	2	390	20	3	390	20
≥ 60	10	100	20	1	480	22.5	1	480	30

Future will	Overall								
	No. 1 Clinician		No. 2 General practitioner		Overall				
	n	%	n	%					
All ages, years	2722	63.4	1006	23.4	4293				
20–	287	10.5	38	3.8	350				
30–	850	31.2	165	16.4	1135				
40–	660	24.2	213	21.1	996				
50–	494	18.1	273	27.1	900				
≥ 60	431	15.8	317	31.5	912				

The median on-call hours were 0 (range, 0–127) hours for the overall population, 41 (range, 0.5–119) for males and 0 (range 0–126.5) for females.

**Figure 2:**
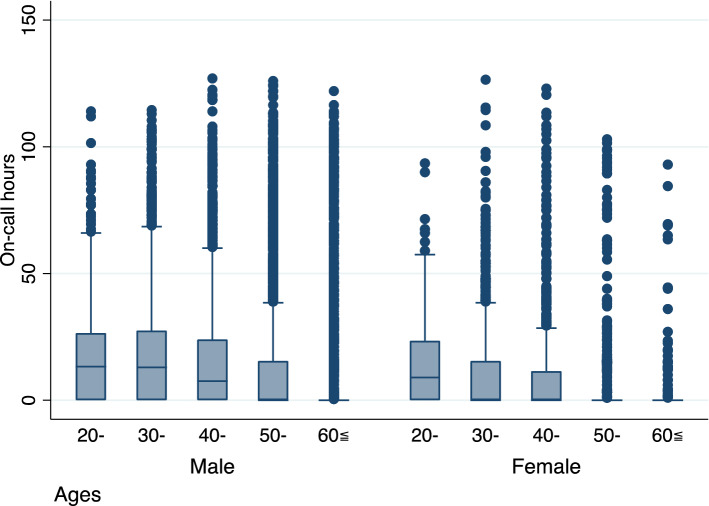
Box-and-whisker diagram of a doctor's weekly on-call hours.

In all the age groups, 1679 (36.0%) doctors (1342 [38.6%] males and 313 [27.9%] females were on call. The proportion of on-call hours decreased with age in both males and females. We summarized the percentages of on-call workers by sex. The results showed sex differences by age (male vs. female): 20s (57.9% vs. 45.2%, respectively; p < 0.001), 30s (58.7% vs. 36.1%, respectively; p < 0.05), 40s (48.6% vs. 23.2%, respectively; p = 0.15), 50s (32.1% vs. 17.25%, respectively; p < 0.05), and ≥ 60s (17.3% vs. 8.4%, respectively; p < 0.001) (Fig. 2).

In the univariate analysis (Table 3), physicians' working hours were statistically associated with age, sex, department, hospital beds, childbirth/childcare experience, and specialist qualification (p < 0.001). In particular, the working hours of female physicians were affected by (1) childbirth and childcare experience (p < 0.001, 95% CI − 10.3 to − 8.4, with “none” as reference) and (2) specialist qualification (p < 0.001, 95% CI − 3.5 to − 1.4, with “none” as reference).

**Table 3:** Univariate analysis of the factors determining the weekly working hours of male and female physicians.

Weekly working hours (h)	Male				Female				Overall			
	Coefficient	p-values	95% confidence interval		Coefficient	p-values	95% confidence interval		Coefficient	p-values	95% confidence interval	
**Ages, years**												
20–	Ref											
30–	− 3.7	p < 0.001*	− 5.0	− 2.4	− 7.8	p < 0.001*	− 9.4	− 6.1	− 5.1	p < 0.001*	− 6.1	− 4.0
40–	− 6.8	p < 0.001*	− 8.1	− 5.5	− 12.1	p < 0.001*	− 13.8	− 10.4	− 7.8	p < 0.001*	− 8.9	− 6.8
50–	− 10.6	p < 0.001*	− 11.9	− 9.3	− 10.3	p < 0.001*	− 12.2	− 8.5	− 9.8	p < 0.001*	− 10.9	− 8.8
60–	− 17.3	p < 0.001*	− 18.6	− 16.0	− 14.3	p < 0.001*	− 16.4	− 12.1	− 16.0	p < 0.001*	− 17.0	− 14.9
**Sex**												
Male									Ref			
Female									− 3.4	p < 0.001*	− 4.0	− 2.8
**Place of work**												
Urban and metropolitan	Ref				Ref				Ref			
Rural	− 0.1	0.82	− 1.0	0.8	0.8	0.32	− 0.8		0.3	0.54	− 0.6	1.1
**Department**												
Internal medicine	Ref				Ref				Ref			
Surgeon	3.6	p < 0.001*	2.7	4.5	7.3	p < 0.001*	4.8	9.8	4.1	p < 0.001*	3.3	5.0
Obstetrics and gynecology	− 1.3	0.12	− 2.9	0.3	0.0	0.98	− 2.0	2.0	− 1.5	p < 0.05***	− 2.8	− 0.2
Pediatrics	− 1.8	p < 0.01**	− 3.1	− 0.5	− 0.8	0.37	− 2.7	1.0	− 1.8	p < 0.01**	− 2.8	− 0.7
Emergency	3.3	p < 0.01**	0.8	5.7	7.8	p < 0.01**	2.2	13.3	4.0	p < 0.01**	1.7	6.2
Anesthesiology	1.8	p < 0.05***	0.0	3.5	4.4	p < 0.001*	2.2	6.5	2.2	p < 0.01**	0.8	3.5
Psychiatry	− 6.6	p < 0.001*	− 7.9	− 5.4	− 6.5	p < 0.001*	− 8.6	− 4.4	− 6.7	p < 0.001*	− 7.8	− 5.7
Radiology	2.7	p < 0.01**	0.8	4.5	1.5	0.33	− 1.4	4.3	2.2	p < 0.01**	0.6	3.8
Resident	6.7	p < 0.001*	4.4	9.0	7.6	p < 0.001*	4.5	10.7	6.6	p < 0.001*	4.8	8.4
**Hospital category**												
Training core	Ref				Ref				Ref			
Emergency	− 0.4	0.75	− 2.5	1.8	− 0.3	0.85	− 3.8	3.1	− 0.3	0.76	− 2.1	1.6
Maternal and perinatal	2.3	p < 0.05***	0.4	4.2	2.2	0.15	− 0.8	5.1	2.6	p < 0.01**	0.9	4.2
Support at home	2.8	p < 0.01**	0.9	4.6	2.6	p < 0.01**	− 0.3	5.4	2.1	p < 0.05***	0.5	3.7
**Number of beds**												
0–19	− 1.8	0.23	− 4.7	1.1	− 8.4	p < 0.01**	− 13.2	− 3.6	− 3.2	p < 0.05***	− 5.7	− 0.7
20–49	− 5.5	p < 0.001*	− 8.3	− 2.7	− 5.6	p < 0.05***	− 10.6	− 0.6	− 4.9	p < 0.001*	− 7.5	− 2.4
50–100	− 2.3	p < 0.05***	− 4.3	− 0.4	− 4.4	p < 0.01**	− 7.6	− 1.2	− 3.3	p < 0.001*	− 5.1	− 1.6
101–200	− 2.4	p < 0.01**	− 3.9	− 0.9	− 1.6	0.24	− 4.3	1.1	− 2.2	p < 0.01**	− 3.5	− 0.8
201–300	Ref				Ref				Ref			
301–400	1.8	p < 0.05***	0.0	3.5	4.0	p < 0.01**	1.1	6.9	2.1	p < 0.01**	0.6	3.6
401–500	3.7	p < 0.001*	1.9	5.5	6.1	p < 0.001*	3.2	9.0	4.1	p < 0.001*	2.6	5.7
≥ 501	1.6	p < 0.05***	0.2	2.9	3.9	p < 0.01**	1.6	6.1	1.9	p < 0.01**	0.7	3.1
**Side job**												
Full time	Ref				Ref				Ref			
Part time	− 0.8	p < 0.01**	− 1.8	0.1	2.6	p < 0.05***	0.2	4.9	1.9	p < 0.001*	1.1	2.8
Self-employed	− 3.6	p < 0.001*	− 5.1	− 2.2	− 0.6	0.52	− 2.6	1.3	− 1.8	p < 0.01**	− 3.0	− 0.7
Unemployed	− 1.8	p < 0.001*	− 2.6	− 1.1	4.9	p < 0.001*	2.5	7.2	1.1	p < 0.001*	0.5	1.8
**Childbirth and childrearing**												
Possession	− 0.40	0.19	− 0.2	0.99	− 9.3	p < 0.001*	− 10.3	− 8.4	2.4	p < 0.001*	− 2.9	− 1.9
None	Ref				Ref				Ref			
**Specialist qualification**												
Possession	− 0.44	0.20	− 1.1	0.22	− 2.45	p < 0.001*	− 3.51	− 1.40	− 0.88	p < 0.05***	− 1.45	− 0.32
None	Ref				Ref				Ref			

*p < 0.001, **p < 0.01, ***p < 0.05.

In the multivariate analysis (Table 4), physician work hours were associated with sex (coefficient − 7.4, 95% CI − 8.3 to − 6.5, with “man” as reference), childbirth/childcare experience (coefficient − 2.2, 95% CI − 2.9 to − 1.4, with “possession” as reference), and specialty qualification (coefficient − 4.0, 95% CI − 5.0 to − 3.0, with “possession” as reference).

**Table 4:** Multivariate analysis of factors determining weekly working hours for male and female physicians.

Weekly working hours (h)	Coefficient	p value	95% confidence interval	
**Ages, years**				
20–	Ref			
30–	− 5.6	p < 0.001*	− 7.4	− 3.8
40–	− 7.4	p < 0.001*	− 9.3	− 5.5
50–	− 9.7	p < 0.001*	− 11.6	− 7.8
≥ 60	− 18.8	p < 0.001*	− 20.8	− 16.9
**Sex**				
Male	Ref			
Female	− 7.4	p < 0.001*	− 8.3	− 6.5
**Department**				
Internal medicine	Ref			
Surgeon	3.6	p < 0.001*	2.5	4.7
Obstetrics and gynecology	− 2.4	p < 0.001*	− 3.9	− 0.8
Pediatrics	− 2.2	p < 0.001*	− 3.5	− 0.9
Emergency	0.8	p < 0.001*	− 1.9	3.5
Anesthesiology	− 2.7	p < 0.001*	− 4.4	− 1.1
Psychiatry	− 8.2	p < 0.001*	− 9.5	− 7.0
Radiology	− 1.4	0.16	− 3.3	0.5
Resident	− 4.2	p < 0.001*	− 6.7	− 1.7
**Childbirth and childrearing**				
Possession	− 2.2	p < 0.001*	− 2.9	− 1.4
None	Ref			
**Specialist qualification**				
Possession	Ref			
None	− 4.0	p < 0.001*	− 5.0	− 3.0

*p < 0.001.

In Table 5, we showed the efforts to work without turnover. The total number of valid responses was 4800 (2265 and 2535 in the groups with and without childcare experience, respectively), consisting of 12 items including reduced overtime work (466 vs. 417, p < 0.05), leave after shift change (187 vs. 280, p < 0.05), vacation promotion (269 vs. 184, p < 0.001), and creation of a system to prevent career delays (82 vs. 137 p < 0.05). There was a significant statistical difference between the two groups.

**Table 5:** Efforts to work without leaving the workforce during childcare (overall).

	Leaving a job				Total	p value*
	Yes		No			
	n	%	n	%		
Effort	2265		2535		4800	
Reduction of overtime work	466	20.6	417	16.4	883	p < 0.05**
Shift personnel	356	15.7	417	16.4	773	p < 0.05**
Increased salary	261	11.5	318	12.5	579	p < 0.01****
Leave after shift change	187	8.3	280	11.0	467	p < 0.001***
Vacation promotion	269	11.9	184	7.3	453	p < 0.001***
Share of other occupations	220	9.7	204	8.0	424	0.29
Babysitter	174	7.7	227	9.0	401	p < 0.05**
Relief of work benefits	139	6.1	163	6.4	302	0.08
Short time to advance	96	4.2	82	5.0	178	0.19
No career delay	82	3.6	137	5.4	219	p < 0.001***
In-hospital childcare facilities	15	0.6	17	0.7	33	0.42

*p-values for the chi-square test for comparisons between the “Yes” and “No” groups of leaving a job (one-sided Fisher's exact).

**p < 0.05, ***p < 0.001, ****p < 0.01.

## Discussion

This study showed that physician consultation times decreased with age for both male and female (Table 2). In Japan, working hours tend to decrease in other occupations, regardless of the type of work^16^; however, they are still high for doctors^17^*.* We have presented the working hours of Japanese physicians. In many countries around the world, it is common for physicians, excluding residents, to work longer hours^10,11^. In Western countries, there are regulations regarding maximum working hours for residents. However, there are no regulations regarding working hours for physicians*.* In Europe and the United States, the problem of doctors (including residents) working long hours became apparent from the 1990s to the 2000s^18,19^. As a result, the working hours were regulated. In Japan, guidelines on working hours for physicians are scheduled to be issued in 2024, more than 20 years later than that in Western countries. However, the current United States study showed that treatment outcomes do not change, regardless of whether residents' working hours are restricted or not^20,21^. Long working hours are an important factor to be considered under labor management because they can lead to burnout and a decline in job performance due to fatigue and lack of concentration^22–26^. While working hours are necessary for self-improvement as part of professional training, too long working hours leave little time for off-job training^27^. The prevalence of burnout among physicians is high globally, and it is important to prevent it. In particular, burnout is strongly associated with a decrease in physicians' motivation to work and learn. As a result, burnout syndrome reduces physicians' work and study time, which has a negative impact on patients' life expectancy. Therefore, more research should be conducted worldwide in the future not only on physician labor, but also on professional demotivation, depression, and burnout.

The present study showed that female physicians worked shorter hours than their male counterparts. This can be attributed to the fact that their partners were doctors (619 women, 63.9% vs. 598 men, 26.8%; p < 0.001) and that they were working full time (1334 women, 74.7% vs. 2629 men, 80.8%), as shown in Table 1. In previous studies^27–30^, working hours were thought to be affected only by childbirth and childcare experience, however, this study proved that they were greatly affected by the occupation and working style of their partner. To support the generation with childcare experience, it is socially important to create a system that allows them to continue working without leaving the workforce.

Table 5 shows that there was a significant statistical difference in the intention to continue working without leaving the workforce between those who had experienced childcare and those who had not. There was a significant difference between the two groups.

Therefore, we believe that clarifying the difference in intentions between the two groups can serve as a basis for the development of social systems. Buddenberg et al.^31^ reported that females become less career-oriented and more part-time oriented after raising children. To date, no study has reportedly compared the intentions of female physicians with and without childcare experience^32,33^. Globally, the percentage of female physicians is increasing yearly^34^. As a result, there is a need to improve the social infrastructure to support them. The goal is that society should work together to ensure that female doctors do not perceive childbirth and childcare as having a negative impact on their career development. In other words, the realization of the Sustainable Development Goals in the medical field is synonymous with comprehensive support for working female doctors^35–37^.

Multiple regression analysis revealed that physicians' working hours are associated with age, sex, department, childbirth/childcare experience, and specialist qualifications. Particularly, working hours generally decreased as age increased for both males and females. It is noteworthy that for men, working hours became longer with the acquisition of a medical specialty. Meanwhile, childbirth and childcare were found to shorten working hours for females. In Tables 3 and 4, we highlighted these two points. Childbirth and childcare experience and the availability of specialist qualifications had an impact on the working hours of female doctors. Working hours are an integral part of building a career. The skills required of a physician are not limited to hospital care but include the ability to think comprehensively about patients and their families and to provide them with the best options. The meaning encompassed by career in this paper is a broad concept that includes specialty medicine, obtaining a doctoral degree, and raising a family. Meanwhile, intermittent study and extensive case experiences are essential to acquire the broad cultivation required of physicians.

This study has some limitations. First, this was a cross-sectional study conducted at one point in time during the winter of 2016. This may result in seasonality in the physicians' working hours and work content. As a result, it may not reflect the standard work content throughout the year. Secondly, although the questionnaire was distributed in advance, recall bias may have occurred when respondents filled it out. Third, this survey was conducted in Japan in 2016. Therefore, the working environment for physicians may differ from current working conditions. The regional distribution of physicians and the ratio of men to women have changed due to changes in the social situation in Japan and the changes that have occurred as a result of these changes. In addition, the special situation of the coronavirus pandemic has been added, and the working environment may differ from that of physicians under normal social activities. The working environment during this pandemic has a significant impact not only on the physical situation of physicians, but also on their mental health^38^. Changes in the working environment have a significant impact on physicians' willingness to work, and as a result, their working conditions are changing as of 2022. Fourth, as the target population has become older, their working conditions, family environment, and career aspirations may differ from those of the same age today.

In conclusion, physicians worked excessive hours, and age, sex, department, children, and specialty certification were associated with the length of working hours. Female physicians' working hours were more strongly influenced by their experience with childbirth and childcare and by their medical specialty than male physicians. Society should create a system that allows both males and females to successfully incorporate childbirth and childcare.

## Supplementary Information

Supplementary Tables.

## Data Availability

The authors confirm that the data supporting the findings of this study are available within the article.
