# Supplementary material for: A retrospective observational study analyzing work and study motivation based on the work environment of 15,677 Japanese clinicians in 2016
Source: Sci Rep. 2022 Aug 31;12:14806. doi: 10.1038/s41598-022-19007-9 (PMC9428877; doi:10.1038/s41598-022-19007-9)
Supplement: Supplementary file 1 — Supplementary Tables. [file 41598_2022_19007_MOESM1_ESM.docx]

(Supplementary)

Table 6 Efforts to work without leaving the workforce during childcare (male)

|  | Leaving a job | | | |  |  |
| --- | --- | --- | --- | --- | --- | --- |
|  | Yes | | No | | Total |  |
|  | n | % | n | % |  | p value* |
| Effort | 1,585 |  | 1,937 |  | 3,522 |  |
| Reduction of overtime work | 350 | 22.1 | 361 | 18.6 | 711 | p<0.05** |
| Shift personnel | 233 | 14.7 | 338 | 17.4 | 571 | p<0.05** |
| Increased salary | 175 | 11.0 | 224 | 11.6 | 399 | 0.35 |
| Leave after shift change | 151 | 9.5 | 237 | 12.2 | 388 | 0.01 |
| Vacation promotion | 195 | 12.3 | 145 | 7.5 | 340 | p<0.001*** |
| Share of other occupations | 135 | 8.5 | 155 | 8.0 | 290 | 0.33 |
| Babysitter | 121 | 7.6 | 164 | 8.5 | 285 | 0.22 |
| Relief work benefits | 100 | 6.3 | 114 | 5.9 | 214 | 0.34 |
| Short time to advance | 60 | 3.8 | 94 | 4.9 | 154 | 0.08 |
| No career delay | 56 | 3.5 | 91 | 4.7 | 147 | 0.06 |
| In-hospital childcare facilities | 9 | 0.6 | 14 | 58.3 | 23 | 0.36 |

*p-values for chi-square test for comparisons between “Yes” and “No” groups of leaving a job (1-sided Fisher's exact)

**p<0.05, ***p<0.001

Table 7 Efforts to work without leaving the workforce during childcare (female)

|  | Leaving a job | | | |  |  |
| --- | --- | --- | --- | --- | --- | --- |
|  | Yes | | No | | Total |  |
|  | n | % | n | % |  | p value* |
| Effort | 680 |  | 598 |  | 1,278 |  |
| Shift personnel | 123 | 18.1 | 124 | 20.7 | 247 | 0.18 |
| Reduction of overtime work | 116 | 17.1 | 56 | 9.4 | 172 | p<0.001*** |
| Increased salary | 86 | 12.6 | 94 | 15.7 | 180 | 0.1 |
| Share of other occupations | 85 | 12.5 | 49 | 8.2 | 134 | 0.01 |
| Vacation promotion | 74 | 10.9 | 39 | 6.5 | 113 | p<0.01** |
| Babysitter | 53 | 7.8 | 63 | 10.5 | 116 | 0.07 |
| Relief work benefits | 39 | 5.7 | 49 | 8.2 | 88 | 0.06 |
| Leave after shift change | 36 | 5.3 | 43 | 7.2 | 79 | 0.11 |
| No career delay | 26 | 3.8 | 46 | 7.7 | 72 | p<0.05** |
| Short time to advance | 36 | 5.3 | 32 | 5.3 | 68 | 0.53 |
| In-hospital childcare facilities | 6 | 70 | 3 | 30 | 9 | 0.32 |

*p-values for chi-square test for comparisons between “Yes” and “No” groups of leaving a job (1-sided Fisher's exact)

**p<0.05, ***p<0.001
